# Sleep disturbance among Chinese survivors of childhood sexual abuse: associations with perceived discrimination and rumination

**DOI:** 10.3389/fpsyt.2025.1431839

**Published:** 2025-02-20

**Authors:** Wan Wang, Xi Wang

**Affiliations:** ^1^ Henan Provincial Medical Key Lab of Child Developmental Behavior and Learning, Department of Child Developmental Behavior, The Third Affiliated Hospital of Zhengzhou University, Zhengzhou, China; ^2^ College of Computer Science and Electronic Engineering, Hunan University, Changsha, China

**Keywords:** sleep disturbance, childhood sexual abuse, perceived discrimination, rumination, Chinese

## Abstract

**Background:**

Although childhood sexual abuse (CSA) has been widely recognized for its association with sleep disturbance (SD) in adulthood, little is known about its associations with perceived discrimination (PD) and rumination. This study seeks to build upon existing literature by examining the impact of CSA on adult SD within a Chinese survivor sample while also investigating the mediating effects of PD and rumination on this association.

**Methods:**

A total of 1,210 respondents completed the Childhood Sexual Abuse Questionnaire, the Inventory of Depressive Symptomatology Self Report, the Perceived Discrimination Scale, and the Ruminative Response Scale.

**Results:**

Participants with CSA experiences reported higher scores of SD and greater rates of insomnia and hypersomnia than those without such experiences (47.79% vs. 29.30%; 20.71% vs. 9.46%). CSA, SD, PD, and rumination were positively related with each other. CSA could predict SD not only directly but also indirectly through the mediating effects of PD and rumination.

**Limitations:**

Due to the cross-sectional design, the study is unable to demonstrate causality but, rather, that there exist important associations that are worth further investigation.

**Conclusions:**

This study examined a sample of Chinese survivors of CSA to establish the link between CSA and adult sleep disturbances (SD). Our findings not only confirm this association but also shed light on the intermediary roles of PD and rumination in shaping this relationship. It suggests that interventions designed to mitigate the sense of discrimination and to foster the adoption of positive coping strategies could be instrumental in enhancing the sleep quality of those who have been sexually abused in their childhood.

## 1 Introduction

As a critical public health issue, childhood sexual abuse (CSA) refers to the involvement of children in sexual activities that they do not fully understand, are unable to give informed consent to, for which they are not developmentally prepared, or that contravene the societal norms (1). CSA is relatively common with prevalence rates of 20%–25% for women and 5%–15% for men (2). Data from published research covering 6,834 Chinese college students reported that the rate of CSA was 20.76%, with men being higher than women (24.23% vs. 18.10%) (3). A survey among Chinese middle school students showed that the prevalence rate of sexual abuse was 25.5% (4).

A multitude of studies have demonstrated that CSA is correlated with a heightened vulnerability to adverse health consequences in later life (5, 6), with sleep disturbance (SD) being among the most notable of these issues (7, 8). Therefore, a more profound comprehension of the relationship between CSA and SD is essential for the prevention, assessment, and treatment of sleep-related issues.

Childhood trauma, particularly CSA, has been consistently linked to sleep problems in adulthood as evidenced by a wealth of research (9–11). Studies have shown that the effects of CSA on sleep can be long-lasting, extending into adulthood (8, 12, 13). CSA survivors are often at a higher risk of experiencing difficulties in falling asleep or maintaining sleep, have lower sleep efficiency, have increased occurrences of nightmares, and have restless sleep (8, 14).—for instance, a longitudinal study involving 533 adult survivors of CSA revealed that the prevalence rate of high insomnia symptoms was as high as 30.6% (13). Additionally, a study focusing on women sexually abused 10 years ago indicated that they reported more SD, identifying such as a long-term consequence of CSA (15). Furthermore, trauma-related psychological problems such as anxiety and depression, which can arise later in life, may then also exacerbate existing SD in CSA survivors (16). However, research confirming the association between CSA and sleep problems among Chinese populations is limited (9). Therefore, it is imperative to further explore the potential pathways linking CSA and SD, especially among Chinese CSA survivors.

Perceived discrimination may indeed serve as a critical link between CSA and SD in adulthood. Perceived discrimination (PD) is defined as people’s beliefs or perceptions of negative attitudes, judgment, or unfair treatment directed toward them as members of a specific group (17, 18). The cognitive model of posttraumatic stress disorder suggests that those who experienced traumatic events may overgeneralize from these events, perceiving a range of activities as more dangerous than they really are (19). Early life trauma can contribute to the development of negative beliefs about the world or the self, increasing the likelihood of feeling discriminated against (20, 21). Compared to those without adverse childhood experiences, adults with such experiences were more likely to experience perceived daily discrimination and lifetime discrimination (17). This heightened perception of discrimination may, in turn, exacerbate sleep disturbances. A growing body of research has consistently shown that discrimination negatively impacts sleep (22, 23). Everyday discrimination is significantly associated with poorer sleep quality and greater disturbances (24). While the existing literature has demonstrated the relationship between adverse childhood experiences and PD in adulthood (21, 25), the specific association between CSA and PD has not been thoroughly explored. There is a need to investigate whether PD plays a significant mediating role in the relationship between CSA and SD. One study has examined PD’s role as a mediator of childhood trauma and psychological distress (26), but the effect of PD on the link between CSA and SD remains understudied. Therefore, further research is necessary to establish whether PD could significantly mediate the association between CSA and SD, which could have important implications for developing targeted interventions to improve sleep quality among survivors of CSA.

Rumination, a maladaptive cognitive processing style or coping strategy in response to trauma (19), is characterized by persistent and negative introspection on one’s suffering, the potential causes, and the consequences of unwanted emotions (27). This pattern often emerges following a traumatic experience and may reinforce problematic interpretations of the trauma (19). According to the cognitive model, rumination can directly amplify feelings of nervous tension, dysphoria, or hopelessness, thereby prolonging and intensifying psychological distress and its associated problems (28, 29). Previous studies have indicated that individuals who have been sexually abused in childhood were more prone to ruminate on their feelings of sadness (30, 31), and this on-going rumination was linked to more severe long-term psychological problems (32), including sleep difficulties (33). Given the relationships between CSA, rumination, and sleep problems, it is hypothesized that CSA can significantly predict SD through the mediating effect of rumination.

Furthermore, rumination and PD have been found to be positively associated with each other (29, 34). A study on racial minorities has indicated that rumination mediated the relationship between PD and depressive symptoms (29). As a maladaptive cognitive strategy, rumination can lead to perseverative thoughts about the feeling of discrimination, which, in turn, can trigger mental health problems (28). However, the extent to which rumination might predict SD through the effect of PD among CSA survivors has not yet been revealed. In consideration of prior studies, we propose the hypothesis that PD and rumination may act as linking factors in the association between CSA and SD.

**Figure 1:**
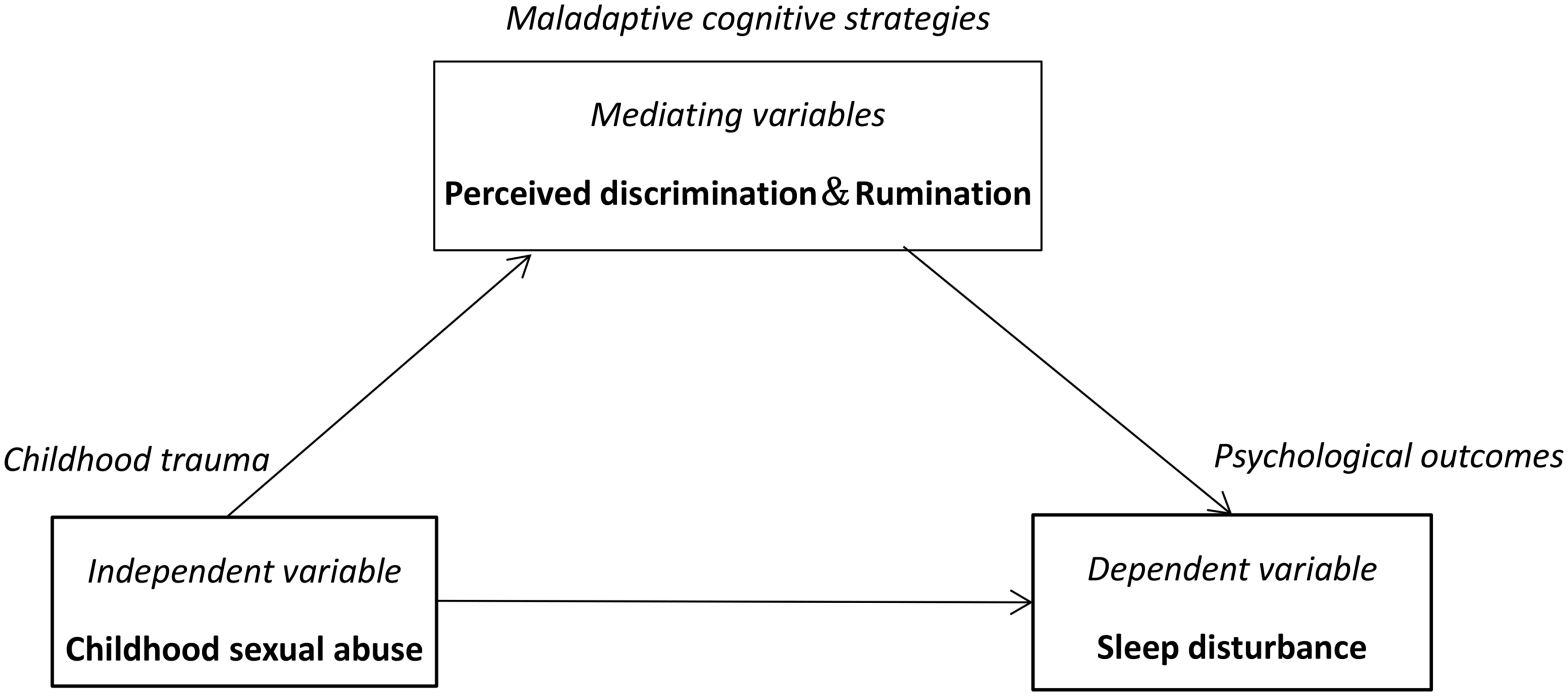
Conceptual framework.

The present study aimed to extend prior research and further elucidate SD among CSA survivors as well as its associations with PD and rumination. More specifically, we want to know the relationship between CSA and adulthood SD and the extent to which PD and rumination mediated this relationship. Drawing from the extant body of research, it is hypothesized that (1) CSA would have positive relations to SD and (2) PD and rumination would show significant mediating effects on the associations between CSA and SD (**Figure 1**).

## 2 Methods

### 2.1 Participants and procedure

The participants were recruited by means of online advertisement, mostly through a web-based platform (https://www.wjx.cn/). After reading the consent form and guidance of the survey, a total of 1,210 respondents (824 men and 386 women) completed this survey. The participants’ ages ranged from 18 to 35 (*M* = 22.77, SD = 3.14). Among them, there were 565 (46.7%) participants with a history of CSA, 508 (42.0%) participants had a left-behind experience, 702 (58.0%) participants were from the countryside, and 310 (25.6%) participants were from single-parent families. This study was conducted in accordance with the Declaration of Helsinki and received ethical approval by the Ethics Committee for Scientific Research of the Third Affiliated Hospital of Zhengzhou University (grant no. 2022-360-01).

### 2.2 Measures

#### 2.2.1 Childhood Sexual Abuse Questionnaire

Sexual abuse history in childhood was assessed using the Childhood Sexual Abuse Questionnaire (CSAQ), a subscale of the Childhood Trauma Questionnaire-Short Form adapted into a Chinese version (35, 36). The CSAQ comprises five items that evaluate the occurrence of sexual abuse during childhood, with each item scored on a five-point scale (1 = never true; 5 = very often true). The participants with a total score of 5 or higher were classified as having a history of CSA. The CSAQ has demonstrated robust psychometric properties in prior studies (5). The Cronbach’s alpha of the CSAQ in the present study was 0.918, indicating high internal consistency.

#### 2.2.2 Inventory of Depressive Symptomatology Self-Report

SD was measured by four sleep-related items derived from the Inventory of Depressive Symptomatology Self Report (IDS-SR): difficulty falling asleep, difficulty maintaining sleep, early awakening, and hypersomnia (37, 38). The participants were asked to rate these items on a four-point Likert scale (0–3) according to their sleep status over the past week. The insomnia total score was calculated as the sum of the scores for the first three items. Based on the scores from these four items, SD can be categorized as insomnia, hypersomnia, and delayed sleep phase. The IDS-SR has been validated by several previous studies (39). The Cronbach’s alpha for the present sample was 0.590.

#### 2.2.3 Perceived Discrimination Scale

The Perceived Discrimination Scale (PDS) was employed to measure perceived discrimination (25). This scale consists of three items, each rated on a four-point scale ranging from 1 (completely not conform) to 5 (completely conform). The participants were asked to report the frequency with which they experienced discriminatory treatment in their daily lives. The PDS has demonstrated good reliability and validity in Chinese samples (40). In the current study, the Cronbach’s alpha for PDS was 0.923.

#### 2.2.4 Ruminative Response Scale

The Ruminative Response Scale (RRS), a 22-item self-report measurement, was utilized to evaluate rumination (27). The RRS comprises three factors: symptom rumination, brooding, and reflective pondering (41). The participants were required to respond on a four-point Likert-scale (1 = almost never; 7 = almost always). Numerous studies have demonstrated that the RRS possesses satisfactory properties (42). The Cronbach’s alpha for RRS was 0.970 in the current study.

### 2.3 Statistical analysis

Descriptive analysis, *t*-test, and bivariate correlations between variables were performed using SPSS 21.0. The mediation effects of PD and rumination on the relationship between CSA and SD were tested using the PROCESS v3.5 (model 6) in SPSS (43). Bias-corrected bootstrap estimates were based on 5,000 samples. The effect was significant when zero was not included in the 95% bootstrap confidence interval.

The structural equation model (SEM) by maximum likelihood estimation (MLE) was conducted using AMOS 23.0. Model fit was estimated based on the following indices: for chi-square (*χ* ^2^) divided by degrees of freedom (CMIN/DF), goodness-of-fit index (GFI), comparative fit index (CFI), adjusted goodness-of-fit index (AGFI), and root mean square error of approximation (RMSEA). The theoretical model was well fitted with CMIN/DF <2, GFI and CFI >0.95, AGFI >0.90, and RMSEA <0.05 (44).

## 3 Results

### 3.1 Group comparisons

The results for group comparisons can be found in **Table 1**. Participants who experienced CSA exhibited significantly higher scores for SD, PD, and rumination compared to those without such experiences. Specifically, individuals with CSA reported higher rates of insomnia (47.79% vs. 29.30%) and hypersomnia (20.71% vs. 9.46%) than their counterparts without CSA. However, there was no significant difference in the rates of DSP between participants who had experienced CSA and those who had not.

**Table 1:** Comparison of frequencies or means on variables of interest.

Variable	Histories of childhood sexual abuse		*x* ^2^/*t*	Gender		*x* ^2^/*t*
	Yes (*n* = 565)	No (*n* = 645)		Male (*n* = 824)	Female (*n* = 368)	
SD	1.97 (1.49)	1.07 (1.40)	10.83***	1.44 (1.50)	1.62 (1.54)	-1.91
Insomnia	270 (47.79%)	189 (29.30%)	43.71***	301 (36.53%)	158 (42.93%)	2.17
Hypersomnia	117 (20.71%)	61 (9.46%)	30.38***	112 (13.59%)	66 (17.10%)	2.58
DSP	11 (1.95%)	11 (1.71%)	0.10	11 (1.33%)	11 (2.85%)	3.78
CSA	11.85 (4.40)	5.00 (0.00)	39.53***	8.39 (4.70)	7.59 (4.19)	2.13*
PD	9.56 (3.19)	6.12 (3.14)	18.86***	9.56 (3.19)	6.12 (3.14)	1.99*
Rumination	57.38 (14.97)	40.93 (15.75)	18.54***	48.99 (16.78)	47.82 (18.38)	1.09

PD, perceived discrimination; CSA, childhood sexual abuse; SD, sleep disturbance; DSP, delay sleep phase.

*p <.05; ***p <.001.

Moreover, male participants reported higher scores of CSA and PD than female participants. However, no significant differences were observed between men and women in terms of SD and rumination as well as the rates of insomnia, hypersomnia, and DSP.

### 3.2 Correlations

The correlations of study variables are presented in **Table 2**. The results indicated that CSA, SD, PD, and rumination were positively related with each other. Age was positively related to CSA, SD, PD, and rumination. Left-behind experience was negatively related to CSA, SD, PD, and rumination. There was no significant correlation between birth place and CSA, SD, PD, and rumination.

**Table 2:** Correlations of study variables (*n* = 1,210).

	CSA	SD	PD	Rumination
CSA	——			
SD	0.35**	——		
PD	0.48**	0.41**	——	
Rumination	0.46**	0.42**	0.83**	——
Gender	-0.06*	0.06	-0.06*	-0.03
Age	0.15**	0.06*	0.14**	0.11**
Birthplace	-0.05	0.00	-0.02	-0.01
Left-behind experience	-0.27**	-0.30**	-0.29**	-0.29**
Single-parent family	-0.31**	-0.24**	-0.28**	-0.29**
M	8.20	1.50	7.73	48.61
SD	4.55	1.51	3.60	17.44

PD, perceived discrimination; CSA, childhood sexual abuse; SD, sleep disturbance.

Left-behind experience refers to the experience of a child growing up without companionship by out-working parents who migrate to cities but leave their children behind in villages.

*p <.05; **p <.01.

### 3.3 Direct and indirect effects

To clarify the effects of CSA on SD through PD and rumination, after controlling for gender, age, birthplace, left-behind experience, and single-parent family, multiple mediation analysis was conducted. The results can be found in **Tables 3**, **4**. The direct effect of CSA on SD was significant (direct effect = 0.151). There was a significant indirect effect of CSA on SD through PD and rumination. This means that CSA could not only predict SD via PD and rumination, respectively (the indirect effect of SD was 0.050 and the ratio of indirect to total effect was 17.99%; the indirect effect of rumination was 0.014 and the ratio of indirect to total effect was 5.04%) but could also predict SD via the multiple mediating effect of PD and rumination (the indirect effect of PD and rumination was 0.063 and the ratio of indirect to total effect was 22.66%).

**Table 3:** Multiple mediation analysis of perceived discrimination and rumination on the relationship between childhood sexual abuse and sleep disturbance.

Dependent variable	Independent variable	*β*	SE	*t*	*p*
SD	CSA	0.28	0.03	9.77	<0.001
PD	CSA	0.40	0.03	15.01	<0.001
Rumination	CSA	0.08	0.02	3.81	<0.001
	PD	0.79	0.02	41.91	<0.001
SD	CSA	0.15	0.03	5.05	<0.001
	PD	0.13	0.05	2.68	<0.01
	Rumination	0.20	0.05	4.35	<0.001

PD, perceived discrimination; CSA, childhood sexual abuse; SD, sleep disturbance.

**Table 4:** Mediation effects of perceived discrimination and rumination between childhood sexual abuse and sleep disturbance.

Paths	LL 95% CI	UL 95% CI	Indirect effect	Direct effect	Total effect	Ratio of indirect to total effect (%)
CSA—PD—SD	0.01	0.09	0.050	0.151	0.278	17.99%
CSA—Rumination—SD	0.01	0.27	0.014			5.04%
CSA—PD—Rumination—SD	0.03	0.09	0.063			22.66%

PD, perceived discrimination; CSA, childhood sexual abuse; SD, sleep disturbance.

**Figure 2:**
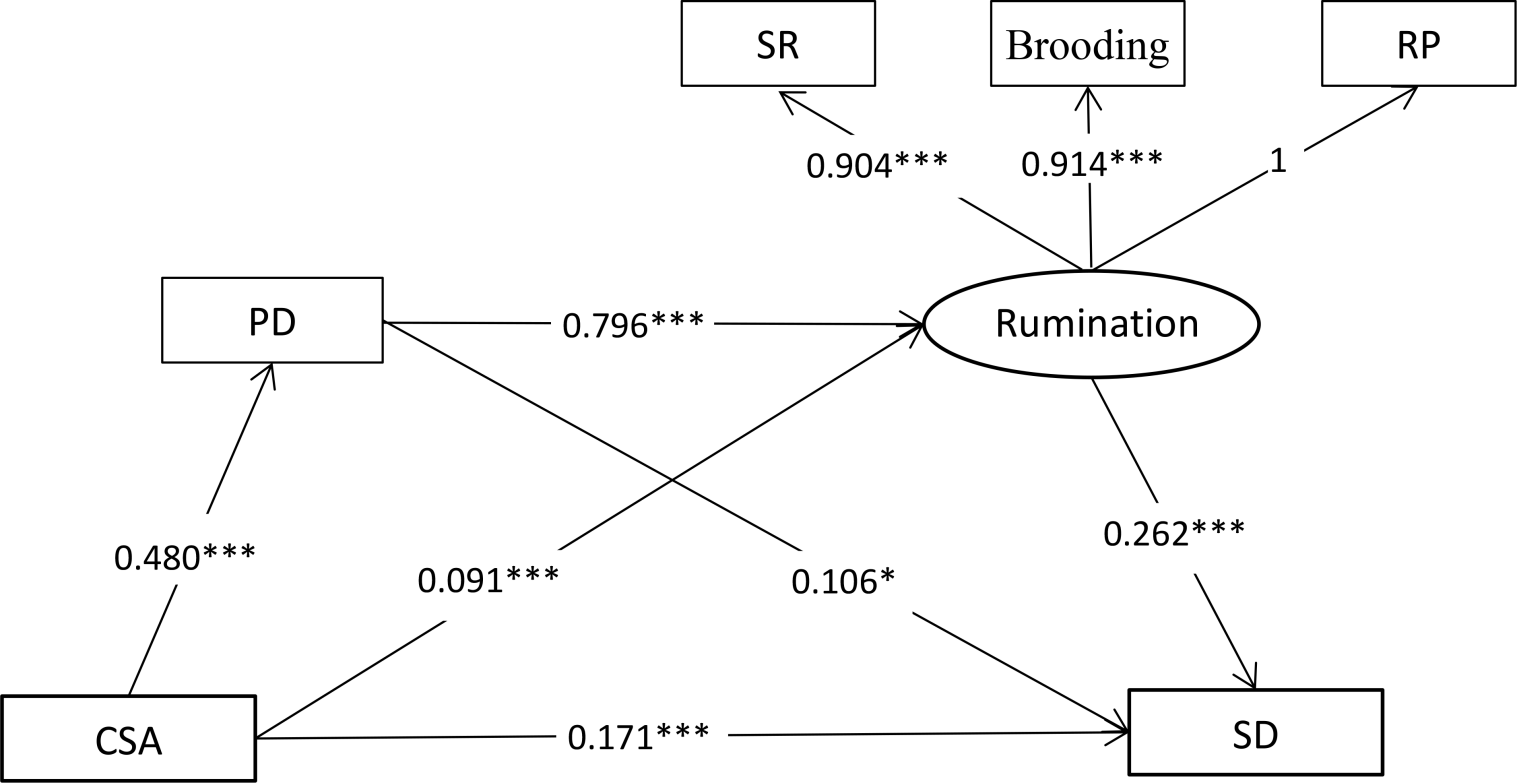
Total model. All coefficients for paths in the structural equation model were standardized. CSA, childhood sexual abuse; SD, sleep disturbance; PD, perceived discrimination; SR, symptom rumination; RP, reflective pondering. *p < .05; ***p < .001.

In order to further visualize the direct effects and indirect effects of CSA on SD through PD and rumination, a total model was performed by using AMOS 21.0. The indices indicated a well fit for the data (CMIN/DF = 3.174, GFI = 0.996, CFI = 0.998, AGFI = 0.982, RMSEA = 0.042). The standardized path coefficients of the total model are displayed in **Figure 2**.

## 4 Discussion

Based upon previous literature on the association between adverse childhood experiences and sleep problems, the current study focused on SD among Chinese CSA survivors and further explored its associations with PD and rumination. As hypothesized, a significant relationship was observed between CSA and SD. Additionally, PD and rumination were found to exert significant mediating effects on the association between CSA and SD.

Our study found that participants with CSA experiences reported higher scores of SD and greater rates of insomnia and hypersomnia than those without such experiences. This aligned with previous research indicating that a higher adverse childhood experience score or childhood trauma score was associated with lower sleep efficiency, more insomnia symptoms, and poorer sleep indices (e.g., wake after sleep onset, sleep onset latency, and sleep quality) (16). Steine et al. (13) pointed out that 30.6% CSA survivors experienced severe insomnia symptoms. Our findings, based on a sample of Chinese CSA survivors, extended prior research by reporting an insomnia prevalence rate of 47.79%, which is notably higher than the previously reported figures (8, 13).

Aligning with a growing body of research (13–15), our study confirmed the positive association between CSA history and SD, with CSA directly predicting adulthood SD. This finding supports our first hypothesis that CSA could have positive relations to SD. Individuals who have experienced childhood traumatic events may be at an increased risk of sleep problems. One potential pathway underlying the link between CSA and adulthood SD is the negative association between SD and sleep-related context, as sexual abuse often occurs during bedtime or in a sleep environment (13). Sleep is inherently confined to unthreatening times and places, yet sexual abuse often occurs during sleeping time (15). Individuals who were sexually abused as children may perceive sleep-related context as threatening, developing long-lasting psychological associations between sleep and fear, anxiety, and wakefulness (45, 46), leading to poor sleep quality in adulthood (15). Another plausible explanation is psychophysiological hyperarousal caused by the hyperactivity of the hypothalamus–pituitary–adrenal cortex (HPA) axis (47), a brain system regulating the sleep/wake cycle (13). Disruption to the stress response and circadian timing systems may also contribute to poor sleep quality following exposure to early life stress (16). Furthermore, individuals abused in childhood are more likely to experience trauma or abuse later in life, and sleep problems may be caused by these more recent trauma or abuse (7). Trauma-related psychological problems (e.g., anxiety, depression) that arise later in life may also exacerbate pre-existing SD in survivors of CSA (16). Additionally, individuals with a history of abuse are more prone to engage in health-impairing behaviors, such as smoking and excessive caffeine or alcohol consumption, which can impact sleep quality directly (48).

To our knowledge, this study is the first to shed light on the mediating effects of PD and rumination on the associations between CSA and SD. Firstly, our findings build upon previous research demonstrating that PD played a mediating role in the associations between childhood trauma and psychological distress (26). This study further extends these insights by indicating PD’s role in the link between CSA and SD. Individuals abused during childhood are more likely to experience negative attitudes, judgment, or unfair treatment from others (17). Early adversities might increase biological, neural, and psychological vulnerability to stress by reducing the hippocampal and amygdala volume, which are potential mechanisms of stress sensitization following exposure to violence (21, 49). These adversities might also foster the development of negative beliefs about the world or the self, increasing the likelihood of perceiving discrimination (20, 21). However, discrimination can induce prolonged threat and chronic stress and potentially lead to the sensitization of the mesolimbic dopamine system and the dysregulation of the hypothalamic–pituitary–adrenal axis, both of which are implicated in various mental health issues, including sleep problems (50). PD has been shown to activate people’s feelings of threat, hyperarousal, or vigilance and even cause psychological distress such as anxiety, depression, anger, or cynical hostility (22). These psychological outcomes may, in turn, lead to poor sleep quality (23, 24).

Secondly, while previous research has explored rumination’s mediating role between PD and depressive symptoms among racial minorities (29), our study extends these findings to a sample of Chinese CSA survivors. We found that rumination mediated the relation between PD and SD, suggesting that CSA may predict SD indirectly through both PD and rumination. Rumination often occurs as a consequence of a traumatic experience, and individuals with a history of CSA were likely to ruminate on their feelings of sadness (30, 31). This maladaptive coping strategy can lead to perseverative thoughts about the feeling of discrimination, which, in turn, causes sleep problems (33).

### 4.1 Implications

The findings of the current study have several notable implications for clinical practice. Firstly, our study lends support to the notion that CSA could have positive relations to SD. As CSA experiences have a detrimental impact on individuals’ mental health, clinicians should be aware that individuals who experienced sexual abuse during childhood may be at an increased risk for sleep problems in adulthood. Secondly, the results of our study provided new evidence to clarify how PD and rumination played mediating effects in associations between CSA experiences and sleep problems. It is noteworthy that these findings supported that the problematic cognitive appraisals and maladaptive coping strategy are critical factors related to SD of CSA survivors. It is crucial for clinicians to consider a wide range of cognitive–behavioral interventions focusing on developing adaptive behavior and positive cognitive strategies. Such interventions may be instrumental in mitigating sleep problems among CSA survivors.

### 4.2 Limitations

Although this study provides new insights into the mediating effects of PD and rumination on the associations between CSA and SD, it is not without limitations. Firstly, our cross-sectional design precludes the establishment of causality between childhood trauma and sleep problems. Therefore, future research employing longitudinal designs is necessary to draw causal conclusions. Secondly, given that different types of childhood trauma may be differently associated with sleep problems, future studies focusing on other types of childhood trauma (e.g., childhood emotional abuse, childhood physical abuse) may enable us to clarify the differences. Thirdly, our study had measurement-specific limitations—for example, because of the retrospective self-assessment style of questionnaires and social desirability of the survey, there might exist recall bias and participants’ bias in the data. This bias can be reduced by maintaining the anonymity of questionnaires in follow-up studies. Lastly, this study did not evaluate any comorbid psychological problems such as anxiety and depression, which may often accompany CSA experiences and may contribute to sleep problems. Therefore, studies in the next step are needed to evaluate and control for such comorbid factors when clarifying the focal associations.

## 5 Conclusions

In conclusion, our study, with a sample of Chinese survivors of CSA, confirms the relationship between CSA and adulthood SD. Specifically, the results of this study are among the first, to our knowledge, to show the mediating effects of PD and rumination in this relationship. These results suggest that interventions aimed at reducing feelings of discrimination and developing positive coping strategies might be beneficial in enhancing the sleep quality of individuals with a history of sexual abuse during childhood.

## Data Availability

The raw data supporting the conclusions of this article will be made available by the authors, without undue reservation.
